# Sacubitril/Valsartan in the Treatment of Resistant Hypertension: Raising Star or Illusion?

**DOI:** 10.3390/jcm11113081

**Published:** 2022-05-30

**Authors:** Marijana Tadic, Cesare Cuspidi

**Affiliations:** 1 Klinik für Innere Medizin II, Cardiology Department, Universitätsklinikum Ulm, Albert-Einstein Allee 23, 89081 Ulm, Germany; 2 Department of Medicine and Surgery, University of Milano-Bicocca, 20126 Milano, Italy; cesare.cuspidi@unimib.it

Sacubitril/valsartan represents the combination that became “*sine qua non*” in the treatment of heart failure with reduced ejection fraction (HFrEF) with significant positive effect on major cardiovascular events [1], and it was included in the guidelines about the therapy in these patients [2]. Soon afterwards the combination of sacubitril and valsartan was proven to have some benefits in patients with HF with preserved ejection fraction (HFpEF) that were not statistically relevant [3]. Even though PARAGON-HF did not show a significant reduction in hospitalization rate due to HF or cardiovascular mortality in patients with HF and an ejection fraction ≥45% [4], it revealed some positive trends in decreasing these adverse outcomes, which is why sacubitril/valsartan has been approved by American Federal Medical Agency (FDA) for treatment of HFpEF in February 2021. Moreover, the first guidelines regarding the specific treatment of HFpEF that were recently released by the American College of Cardiology recognized the importance of sacubitril/valsartan and recommended it as the first-line therapy in HFpEF together with sodium-glucose co-transporter-2 (SGLT2) inhibitors, mineralocorticoid receptor antagonist (MRA), angiotensin converting enzyme inhibitors/angiotensin type I receptor blockers (ACEI/ARB) and diuretics in the treatment of HFpEF [4].

Pluripotential action of sacubitril/valsartan, a first-in-class dual action molecule angiotensin-receptor-neprilysin-inhibitor (ARNI), has recently drawn attention of medical community as a potentially very effective antihypertensive drug in the patients with resistant hypertension [5,6]. The most prevalent comorbidity, present in approximately 90% of HFpEF patients, that might be also considered as the most relevant contributor to HFpEF development is arterial hypertension [7]. The *post hoc* analysis of PARAGON-HF trial showed that 731 HFpEF patients (15.2%) had apparent resistant hypertension and 135 (2.8%) had apparent MRA-resistant hypertension [5]. In patients with resistant hypertension the composite outcome that included number of total hospitalizations for HF and death from cardiovascular causes was significantly higher than in patients with well-controlled hypertension [5]. What was more interesting is that the reduction in systolic blood pressure (BP) after 1 and 4 months was greater with ARNI than with valsartan in patients with resistant hypertension (−4.8 and −3.9 mmHg, respectively) and MRA-resistant hypertension (−8.8 and −6.3 mmHg, respectively) [5]. After 4-month treatment almost half of patients (47.9%) with resistant hypertension treated with ARNI had controlled BP, whereas only third of patients (34.3%) treated with valsartan achieved the same result. The difference was even more prominent in patients with MRA-resistant hypertension (43.6% vs. 28.4%, respectively) [5]. Considering the fact that patients have been already treated with the recommended combination of 3 antihypertensive drugs (ACEI/ARB, CCB and diuretics), this additional reduction in systolic BP in patients with resistant hypertension is even more remarkable. Higher reduction in systolic BP among MRA-resistant hypertensive patients might be explained by synergistic and complementary mechanisms of action of the two drugs, with spironolactone targeting sodium and restricting water retention and neprilysin inhibition of natriuretic peptide, as well as possible decrease in sympathetic activation [8]. Sacubitril/valsartan combination reduces arterial stiffness significantly more than ARB, which might be additional reason for beneficial results in patients with resistant hypertension [9].

The other recently published study in patients with mild to moderate hypertension showed that ARNI induced significantly higher systolic and diastolic BP reduction in comparison with ARB (olmesartan) after only 8 weeks of treatment [10]. Patients treated with ARNI achieved a higher BP control rate comparing to those who were treated with olmesartan. However, the authors only proved that ARNI is a very potent antihypertensive drug, but not in the patients with resistant hypertension. The PARAMOUNT trial revealed that the 12-week systolic BP reduction was significantly greater in the ARNI group in comparison with the valsartan group (−29.3 vs. −22.9 mmHg, respectively) [11], which is even more interesting as ARNI involves the same ARB molecule (valsartan), besides neprilysin-inhibitor (sacubitril). These results emphasized the importance of sacubitril on additional BP reduction and were the main hypothesis for the *post hoc* studies in HFpEF patients which followed [5].

A small study that included patients with resistant hypertension who are on hemodialysis showed significant reduction in systolic and diastolic BP over 12-week period of follow-up (−22.4 mmHg and −8.3 mmHg, respectively) [6]. The largest BP reduction was achieved in the first 4 weeks of treatment and afterwards the trend of reduction maintained, but to a lesser extent. The authors were focused on the performance of the left ventricle (LV) over the course of this study and reported that even though no significant difference in LV structure and function was noticed during this study, there was a significant improvement in parameters of myocardial work (myocardial work index and constructive work) after 12 weeks of therapy [6]. These mechanical changes, together with biochemical improvement (reduction in pro-BNP) might cause better outcome reported in HFpEF patients. Nevertheless, this is a very small study (*n* = 18) and can only serve for making hypothesis for future studies in patients with resistant hypertension that is very prevalent among hemodialysis patients, whose therapy is particularly challenging due to restrictions or necessary adjustments for many antihypertensive medications.

Several mechanisms have been proposed to explain the positive relationship between valsartan/sacubitril combination and hypertension and particularly resistant hypertension that represents a real clinical challenge for everyday practice. Neprilysin inhibition dependent on biologically active natriuretic peptides (ANP), and their binding with particulate guanylate cyclase (GC)/cyclic guanylate monophosphate (cGMP)-coupled receptors, that induce vasodilation, decrease in vascular stiffness, reduction in oxidative stress, diuresis, natriuresis, balance of the renin–angiotensin–aldosterone system (RAAS), the sympathetic nervous system (SNS) and endothelin and vasopressin [8]. The combination of valsartan and neprilysin also reduces the free radicals, level of tumor necrosis factor alpha and proinflammatory cytokines that are responsible for RAAS/SNS overactivation, fibrosis, vascular and microvascular dysfunction and LV diastolic dysfunction.

From clinical perspective ARNI is very useful in patients with resistant hypertension and HFpEF because of increased cardiovascular mortality in these patients despite lower median pro-BNP values in comparison to HFpEF patient without resistant hypertension, which is quite unexpected finding considering the fact that the main paradigm in HF claims that higher pro-BNP has been always related with worse outcome [5]. The recent results are challenging this theory and raising the question about the importance of comorbidities, and resistant hypertension at the first place, on cardiovascular outcome in HFpEF. It seems that new data provided new piece of the puzzle that we were not even aware of and might be very important for the whole picture of complex entity such as HFpEF. The fact that the proportion of responders was higher in patients with MRA-resistant hypertension reveals complementary mechanisms of action between MRA and ARNI, which could be essential for treatment of patients with resistant hypertension with or without HFpEF. Considering the fact that other frequent comorbidities in HFpEF, such as diabetes, obesity, renal dysfunction or amyloidosis were not separately investigated, it is possible that ARNI does not have such important influence as in HFpEF patients with resistant hypertension.

In conclusion, valsartan/neprilysin has not been approved for treatment of resistant hypertension yet, and further studies and *post hoc* analysis are warrantied. However, existing data are encouraging as they show that we finally have a new drug that can help us in the treatment of resistant hypertension.

## Data Availability

Not applicable.
